# Methyl glyoxal increase apoptosis (CASPASE-3 expression) in pre-osteoblast MC3T3E1 cell line via SOD activity

**DOI:** 10.1186/ar3630

**Published:** 2012-02-09

**Authors:** Izaak Zoelkarnain Akbar, Handono Kalim, Djoko Wahono Soeatmadji, Mohammad Hidayat

**Affiliations:** 1 Department of Orthopaedic, Ulin General Hospital, Faculty of Medicine, Lambung Mangkurat University, Banjarmasin, Indonesia; 2 Department of Internal Medicine, Saiful Anwar General Hospital, Faculty of Medicine, Brawijaya University, Malang, Indonesia; 3 Department of Orthopaedic, Saiful Anwar General Hospital, Faculty of Medicine, Brawijaya University, Malang, Indonesia

## Background

Increased advanced glycation end (AGE) products have been reported to be an important cause of increased osteoblast apoptosis in osteoporosis [1,2]. Methylglyoxal (MG) is a reactive dicarbonyl compound endogenously produced mainly from glycolytic intermediates. The involvement of specific reactive oxygen spesies (^•^OH/ H_2_O_2 _/ ^•^O_2_^-^) in increased apoptosis (caspase-3 expression) caused by methyl glyoxal exposure in osteoblast still speculative[3,4]. The aim of our study is to assess the role of specific reactive oxygen species signalling on the effect of MG as an AGE on increased caspase-3 expression in pre-osteoblast.

## Materials and methods

Pre-osteoblast MC3T3E1 cell line was obtained from American Type Culture Cell. Caspase-3 expression in the cells were assayed in basal condition and after the cells exposed with methyl glyoxal on dose 5 μM for 6 hours incubation. Diethylthiocarbamoic acid, mercaptosuccinate, or deferoxamine was added in the culture media to block specific reactive oxygen species signalling for the development of osteoblast apoptosis. The caspase 3 expression were assesses from each different groups of preosteoblast culture: preosteoblast exposed to nothing, preosteoblast exposed to methyl glyoxal, preosteoblast exposed to diethylthiocarbamoic (SOD blocker), exposed to mercaptosuccinate (glutathion peroxidase blocker) and exposed to deferoxamine (Fe^++ ^blocker); and osteoblast exposed to methyl glyoxal and diethylthiocarbamoic, or mercaptosuccinate, or deferoxamine. The result were analyzed using Kruskall Wallis test with p < 00.5 significant.

## Results

Our study showed that MG significantly increased caspase3 expression (apoptosis) of osteoblast. Expression of caspase3 in osteoblast were significantly highest when the cells exposed to SOD blocker compare with when the cells exposed to GSH and Fe^++ ^blocker whether the cells exposed to MG. Hydroxyl radical increase caspase-3 expression higher than another reactive oxygen species (^•^OH > H_2_O_2 _>^•^O_2_^-^) in pre-osteoblast MC3T3E1 without exposed methyl glyoxal. The result showed that superoxide radical more dominant in increasing caspase-3 expression than another reactive oxygen species (^•^O_2_^- ^>^•^OH > H_2_O_2_) in pre-osteoblast MC3T3E1 with MG exposure. There is no significant differences regarding the effecfts of GSH and Fe^++^block on osteoblast caspase3 expression.

## Conclusion

The increased osteoblast apoptosis caused by AGE (MG) is mediated by specific reactive oxygen signalling, SOD activation.
